# Trajectories of physical frailty and cognitive impairment in older adults in United States nursing homes

**DOI:** 10.1186/s12877-022-03012-8

**Published:** 2022-04-19

**Authors:** Yiyang Yuan, Kate L. Lapane, Jennifer Tjia, Jonggyu Baek, Shao-Hsien Liu, Christine M. Ulbricht

**Affiliations:** 1 Clinical and Population Health Research PhD Program, Graduate School of Biomedical Sciences, University of Massachusetts Chan Medical School, Worcester, MA USA; 2 Department of Population and Quantitative Health Sciences, University of Massachusetts Chan Medical School, Worcester, MA USA; 3 Formerly: Department of Population and Quantitative Health Sciences, University of Massachusetts Chan Medical School, Worcester, MA USA; 4 Currently: National Institute of Mental Health, National Institutes of Health, Bethesda, MD USA

**Keywords:** Physical frailty, Cognitive impairment, Nursing home, Trajectory, Group-based trajectory model

## Abstract

**Background:**

U.S. nursing homes provide long-term care to over 1.2 million older adults, 60% of whom were physically frail and 68% had moderate or severe cognitive impairment. Limited research has examined the longitudinal experience of these two conditions in older nursing home residents.

**Methods:**

This national longitudinal study included newly-admitted non-skilled nursing care older residents who had Minimum Data Set (MDS) 3.0 (2014–16) assessments at admission, 3 months, and 6 months (*n* = 266,001). Physical frailty was measured by FRAIL-NH and cognitive impairment by the Brief Interview for Mental Status. Separate sets of group-based trajectory models were fitted to identify the trajectories of physical frailty and trajectories of cognitive impairment, and to estimate the association between older residents’ characteristics at admission with each set of trajectories. A dual trajectory model was used to quantify the association between the physical frailty trajectories and cognitive impairment trajectories.

**Results:**

Over the course of the first six months post-admission, five physical frailty trajectories [“Consistently Frail” (prevalence: 53.0%), “Consistently Pre-frail” (29.0%), “Worsening Frailty” (7.6%), “Improving Frailty” (5.5%), and “Consistently Robust” (4.8%)] and three cognitive impairment trajectories [“Consistently Severe Cognitive Impairment” (35.5%), “Consistently Moderate Cognitive Impairment” (31.8%), “Consistently Intact/Mild Cognitive Impairment” (32.7%)] were identified. One in five older residents simultaneously followed the trajectories of “Consistently Frail” and “Consistently Severe Cognitive Impairment”. Characteristics associated with higher odds of the “Improving Frailty”, “Worsening Frailty”, “Consistently Pre-frail” and “Consistently Frail” trajectories included greater at-admission cognitive impairment, age ≥ 85 years, admitted from acute hospitals, cardiovascular/metabolic diagnoses, neurological diagnoses, hip or other fractures, and presence of pain. Characteristics associated with higher odds of the “Consistently Moderate Cognitive Impairment” and “Consistently Severe Cognitive Impairment” included worse at-admission physical frailty, neurological diagnoses, hip fracture, and receipt of antipsychotics.

**Conclusions:**

Findings provided information regarding the trajectories of physical frailty, the trajectories of cognitive impairment, the association between the two sets of trajectories, and their association with residents’ characteristics in older adults’ first six months post-admission to U.S. nursing homes. Understanding the trajectory that the residents would most likely follow may provide information to develop a comprehensive care approach tailored to their specific healthcare goals.

**Supplementary Information:**

The online version contains supplementary material available at 10.1186/s12877-022-03012-8.

## Introduction

Physical frailty, characterized by decreased physical reserve and increased vulnerability to external stressors, and cognitive impairment, manifested as dysfunctions on at least one cognitive domain, are common geriatric conditions. In community-dwelling older adults living in the United States (U.S.), 6% have physical frailty only, 26% have cognitive impairment only, and 9% experience both conditions [1]. Physical frailty and cognitive impairment are more prevalent in nursing home residents [2]. Between 30–85% of older nursing home residents experience physical frailty [3–5], and over 60% have moderate to severe cognitive impairment [2, 3]. Despite the high prevalence and the increased risk for hospitalization, lower quality of life, and mortality associated with physical frailty and cognitive impairment [6–8], limited research has examined the longitudinal experience of these two conditions among older nursing home residents.

Changes in the prevalence of physical frailty and cognitive impairment occur in the first three months of nursing home residence [3]: 23% of older residents who were pre-frail at admission became physically robust while 31% became frail after three months; 20% of those with moderate cognitive impairment at admission were found to experience intact/mild impairment while 23% were found to be severely impaired after three months [3]. These prevalence estimates characterize the overall burden of physical frailty and cognitive impairment in the months after nursing home admission. However, population-level prevalence may not reflect how physical frailty and cognitive impairment progress on an individual level, as older adults may experience distinctive trajectories of physical frailty [9, 10] and cognitive impairment [11]. Furthermore, while categories such as robust vs. prefrail vs. frail and intact/mild vs. moderate vs. severe cognitive impairment have clinical value, they may not adequately capture the dynamic continuum of these conditions [12, 13]. Therefore, exploring older residents’ physical frailty trajectories and cognitive impairment trajectories using continuous measures could provide additional insight on the “natural history” of frailty and cognitive decline. Identifying factors associated with trajectories of physical frailty and cognitive impairment could help identify older residents who may be more likely to improve or be at risk for accelerated decline. Such information could guide appropriate treatments in the nursing home setting.

Physical frailty and cognitive impairment are highly correlated [3, 14–17]. In nursing homes, 70% of older residents who were physically frail also had severe cognitive impairment [3], and those with greater levels of cognitive impairment experienced more indicators of physical frailty [17]. This close interrelationship may reflect a shared neuropathology [16, 18], concomitant yet separate outcomes of the aging process, or “cognitive frailty”, defined as the co-existence of both conditions without overt dementia and other neurological conditions [19, 20], but the underlying mechanism remains unclear [21]. To date, evidence-based disease modifying treatments for cognitive impairment are scant [21]. However, physical frailty is potentially reversible [22–24] and could serve as a promising intervention target [25, 26]. Reducing the progression of physical frailty through physical activity and nutritional approaches [25, 27] may help improve the trajectories of cognitive decline. Evaluating the extent to which trajectories of physical frailty are associated with the trajectories of cognitive impairment in older nursing home residents would further our knowledge on the interrelationship between these two conditions with the hope of providing foundational knowledge to develop tailored care for residents to address both conditions.

This study examined the trajectories of physical frailty and cognitive impairment in a national cohort of U.S. older nursing home residents. We focused on the first six months post admission because this is a critical window during which older residents adjust to clinical care and living environment changes [28, 29]. The objectives were to: (1) identify the trajectories of physical frailty and estimate the association between cognitive impairment, demographic and clinical characteristics at admission and the identified physical frailty trajectories; (2) identify the trajectories of cognitive impairment and estimate the association between physical frailty, demographic and clinical characteristics at admission and the identified cognitive impairment trajectories; (3) quantify the association between the physical frailty trajectories and cognitive impairment trajectories.

## Method

### Data

We used the national data repository for all residents in U.S. Medicaid-/Medicare-certified nursing homes, the Minimum Data Set (MDS) 3.0. The MDS 3.0 assessments are completed at admission, quarterly and annually post-admission to collect data through self-report and/or staff-administered questionnaires on residents’ demographic characteristics, cognitive functioning, mood, behavioral status, physical functioning, bladder and bowel conditions, diagnoses, health conditions, nutritional status, medications and other treatment [30].

### Sample

We included residents who were (1) aged ≥ 65 years at admission; (2) newly admitted (defined as no previous stay in a nursing home in the 90 days before the current admission) between 01/01/2014 and 06/30/2016; (3) had a prognosis of life expectancy ≥ 6 months at admission, determined by answering “no” to the MDS 3.0 question, “Does the resident have a condition or chronic disease that may result in a life expectancy of less than 6 months?” [31, 32]; (4) resided in the nursing home for at least six months; (5) had MDS 3.0 assessments at admission, 3 months (closest assessment to 90 days ± 31 days), and 6 months (closest assessment to 180 days ± 31 days); (6) participated in the Brief Interview for Mental Status (BIMS) on each MDS assessment; (7) did not enter as a skilled nursing facility (SNF) resident. We excluded residents admitted for a SNF stay because they were not expected to reside in the nursing home for a prolonged period of time. The final sample included 266,001 residents. (Supplement Figure S.1) The admission, 3-month, and 6-month assessments were used in the analysis.

### Measures

#### Physical frailty

FRAIL-NH was developed to assess physical frailty in nursing home residents using items readily available in MDS 3.0 [33, 34]. As shown in Supplement Table S.1, each item was scored and the total score determined by summing the individual items (range; 0 to 13) [4]. The continuous FRAIL-NH score was used in the primary trajectory analyses. Additionally, to describe physical frailty at admission, the total FRAIL-NH score was categorized using previously validated cutoffs as robust (score: 0–5), pre-frail (score: 6–7), and frail (score: ≥ 8) [4].

#### Cognitive impairment

The BIMS focuses on temporal orientation and ability to recall and is included on Section C [35] of the MDS 3.0. The BIMS is used to assess the level of cognitive impairment in residents who are able to participate in the cognition assessment. The continuous BIMS score (ranging from 0 to 15) [36] was used in the primary trajectory analysis. Additionally, to describe cognitive impairment at admission, the BIMS score was categorized into intact/mild impairment (score: 13–15), moderate impairment (score: 8–12), and severe impairment (score: 0–7) using previously validated cutoffs [36].

#### Demographic and clinical characteristics

The following characteristics were drawn from the admission assessment: age (65- < 75 years; 75- < 85 years; ≥ 85 years), sex (male; female), race/ethnicity (non-Hispanic White; racial/ethnic minority, including American Indian or Alaska Native, Asian, Black or African American, Native Hawaiian Other Pacific Islander, multi-racial, and Hispanic or Latino of any race), location of the nursing homes (urban; rural), sources of admission [community; acute hospital; other sources, including another nursing home/swing bed, psychiatric hospital, inpatient rehabilitation facility, intellectual disabilities and developmental disabilities facilities, hospice, and other unspecified facilities], active diagnoses, any presence of pain (yes/no), and receipt of medications in the 7 days before the MDS 3.0 assessment [antipsychotics (yes/no), antianxiety medications (yes/no), antidepressants (yes/no), or hypnotics (yes/no)]. Active diagnoses included cancer, heart failure, hypertension, diabetes mellitus, Alzheimer’s disease, cerebrovascular accident/transient ischemic attack(TIA)/stroke, non-Alzheimer’s/other dementia [vascular or multi-infarct dementia, mixed dementia, frontotemporal dementia (e.g., Pick’s disease), and dementia related to stroke, Parkinson’s or Creutzfeldt-Jakob diseases], multiple sclerosis, Parkinson’s disease, seizure disorder/epilepsy, arthritis, osteoporosis, hip fracture, other fracture, asthma/chronic obstructive pulmonary disease (COPD)/chronic lung disease, anxiety disorder, and depression.

### Statistical analysis

Sample characteristics at admission were first described in percentages.

To address the first two study objectives, two sets of group-based trajectory models (GBTM) were fitted respectively for: 1) physical frailty (using the continuous total FRAIL-NH score in the censored normal distribution at admission, 3 months, and 6 months), and 2) cognitive impairment (using the continuous BIMS score in the censored normal distribution at admission, 3 months, and 6 months), with time measured by the length of stay (in days) when each of the respective assessments occurred. We then estimated the association between sample characteristics at admission and the identified trajectories. We followed the three-step model building process recommended by Nagin [37] and reported the results according to the GRoLTS-Checklist [38].

#### Step 1

We first identified the trajectories. We used the same approach for the physical frailty and cognitive impairment. GBTM with two to six trajectory groups were fitted to the data with all groups in quadratic shape, the highest possible order for 3 time points. We compared the fit statistics and graphic depictions of these models to see if adding another trajectory identified a new unique experience or if it overlapped with the existing ones. Fit statistics included (1) Bayesian information criterion (BIC): the model with the greater BIC was preferred; (2) Group average posterior probability (AvePP) of assignment: An AvePP >0.7 for all trajectory groups was indicative of good certainty of group assignments; (3) Odds of correct classification (OCC): A model was considered to have high assignment accuracy with OCC>5 for all trajectory groups. From these models, predicted values of FRAIL-NH (or BIMS) scores were graphed as solid lines to depict the trajectories with 95% confidence bands (shown with dashed lines). After we determined the optimal number of trajectories, we optimized the shapes of each trajectory (quadratic or linear) to further improve the model fit. The model that best fit the data was identified to represent the trajectories of physical frailty or the trajectories of cognitive impairment. We assigned qualitative labels to each trajectory guided by validated cutoffs for physical frailty [4] and cognitive impairment [36]. The prevalence of each trajectory was reported for physical frailty trajectories and cognitive impairment trajectories.

#### Step 2

We then selected the residents’ characteristics to be included in each trajectory model. We first “hard-assigned” residents into the trajectory to which they had the highest posterior probability of belonging. The distributions of demographic and clinical characteristics were calculated by the assigned trajectory groups. Then, we used multinomial logistic regression models to estimate and compare the crude and adjusted associations between demographic and clinical characteristics and the assigned trajectory groups. These models helped us identify the final set of characteristics to be included in the GBTMs in Step 3. The Method Supplement provides additional details regarding our modeling approach. Although possible to identify covariates directly in the GBTM, Nagin [37] recommends using conventional multinomial logistic model because it is less computationally demanding. Given our large sample size, we heeded Nagin’s advice.

#### Step 3

We then estimated the association between demographic and clinical characteristics and the trajectories. We included cognitive impairment at admission and the selected demographic and clinical characteristics (Step 2) in the final physical frailty GBTM model (Step 1) to estimate the association between the characteristics and the identified physical frailty trajectories. Similarly, physical frailty at admission and the selected demographic and clinical characteristics (Step 2) were included in the final cognitive impairment GBTM (Step 1) to estimate the association between the characteristics and the identified cognitive impairment trajectories. From these models, we derived adjusted odds ratios (aOR) and corresponding 95% confidence intervals (CI).

To address the third study objective, a dual trajectory model was used. This model allowed us to examine the association between the identified physical frailty trajectories and the identified cognitive impairment trajectories. Three sets of conditional probabilities linked membership across the physical frailty and cognitive impairment trajectory groups [37]. Explicitly, the conditional probabilities were: 1) probability of following each of the physical frailty trajectories conditional on each cognitive impairment trajectory, 2) the probability of following each of the cognitive impairment trajectories conditional on each physical frailty trajectory, and 3) the probability of jointly following a given physical frailty trajectory and a given cognitive impairment trajectory.

The analysis used SAS 9.4 (Cary, NC) with PROC TRAJ for GBTM [39]. We created the figures in R with *ggplot2 *[40].

## Result

### Sample characteristics

Of the 266,001 eligible older residents, nearly half were aged ≥ 85 years, two thirds were women, less than one in five belonged to a racial/ethnic minority group, three quarters entered nursing homes located in urban settings, 35.7% were admitted from the community, and 38.8% from acute hospitals. At admission, 54.1% older adults were frail as measured by the FRAIL-NH scale, and 36.7% had severe cognitive impairment according to BIMS. The top five diagnoses were hypertension (75.9%), Non-Alzheimer’s/Other dementia (42.5%), depression (39.8%), diabetes mellitus (30.2%), and arthritis (29.3%). Pain was documented in over one third of residents. In the past seven days, 19.0% received antipsychotics and 46.9% received antidepressants. (Table 1).

**Table 1:** Characteristics of older adults who were newly admitted to nursing homes as non-skilled nursing facility residents in 2014–2016

	**All**
	**(*n* = 266,001)**
	(%)
**Age (years)**	
65—< 75	20.3
75—< 85	33.9
> = 85	45.8
**Female**	67.3
**Race/ethnic minority**	17.5
**Rural nursing home**	25.6
**Admission source**	
Community	35.7
Acute hospital	38.8
Other sources^a^	25.6
**Physical frailty**^**b**^	
Robust	17.2
Pre-frail	28.7
Frail	54.1
**Cognitive impairment**^**c**^	
Intact/Mild impairment	33.1
Moderate impairment	30.2
Severe impairment	36.7
**Diagnosis**	
Cardiovascular/metabolic	
Heart failure	16.5
Hypertension	75.9
Diabetes Mellitus	30.2
Neurological	
Alzheimer's Disease	13.8
Cerebrovascular accident/Transient ischemic attack/Stroke	11.8
Non-Alzheimer’s/Other dementia ^d^	42.5
Multiple Sclerosis	0.6
Parkinson's Disease	6.3
Seizure disorder/Epilepsy	5.3
Musculoskeletal	
Arthritis	29.3
Osteoporosis	13.6
Hip fracture	2.6
Other fracture	5.8
Cancer	5.8
Asthma/Chronic obstructive pulmonary disease/Chronic lung disease	18.2
Mental health	
Anxiety disorder	23.3
Depression	39.8
**Any presence of pain**	36.6
**Types of psychotropic medications received**	
Antipsychotics	19.0
Antianxiety	18.4
Antidepressant	46.9
Hypnotic	4.0

^a^ Included another nursing home/swing bed, psychiatric hospital, inpatient rehabilitation facility, intellectual disabilities and developmental disabilities facility, long-term care hospitals, hospice, and other unspecified admission sources. ^b^ Measured by FRAIL-NH using previously validated cutoffs: robust (0–5), pre-frail (6–7), and frail (≥ 8).^c^ Measured by BIMS using previously validated cutoffs: intact/mild impairment (13–15), moderate impairment (8–12), and severe impairment (0–7). ^d^ Included non-Alzheimer’s dementia (e.g., vascular or multi-infarct dementia), mixed dementia; frontotemporal dementia (e.g., Pick’s disease), and dementia related to stroke, Parkinson’s or Creutzfeldt-Jakob disease

### Physical frailty trajectories in the first six months of nursing home residence and associated characteristics

We first examined the GBTMs with two to six trajectory groups where all trajectories were estimated as quadratic (Step 1). All models showed good certainty and accuracy of group assignment. (Supplement Table S.2a) The model with five groups captured distinctive trajectories that were not identified in models with two to four groups and was more parsimonious than the six-group model, which, despite a greater BIC, identified several overlapping trajectories. (Supplement Figure S.2a) Therefore, the five-group model was selected for further adjustment in the shape parameters to improve model fit. For this five-group GBTM, 32 models with each trajectory set to either linear or quadratic shape were compared. The final model identified had a quadratic trajectory for group 1 to 4 and linear trajectory for group 5.

**Fig. 1:**
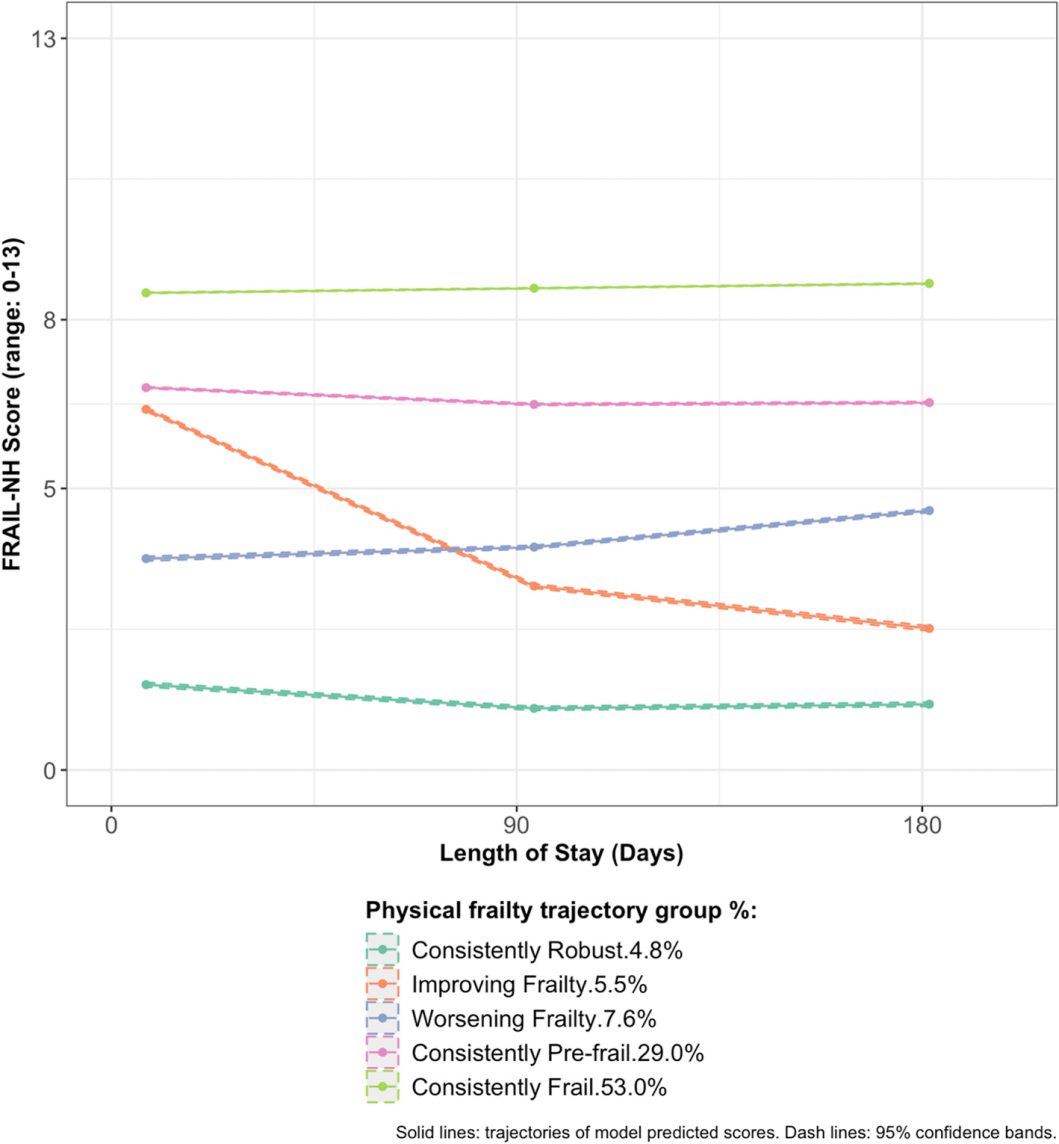
Physical frailty trajectories in the first six months of nursing home stay in older adults newly admitted to nursing homes as non-skilled nursing facility residents in 2014–2016

As shown in Fig. 1, in the first six months post-admission, 5.2% of older residents were consistently physically robust (“Consistently Robust”), 5.5% showed an improvement over time (“Improving Frailty”), 7.6% showed a tendency to be worsening (“Worsening Frailty”), 29.0% consistently following a pre-frail trajectory (“Consistency Pre-frail”), and 53.0% consistently followed a frail trajectory (“Consistently Frail”).

Next, in Step 2, each resident was assigned to the physical frailty trajectory group to which they had the highest posterior probability of belonging. As shown in Supplement Table S.3a, about one in every four older residents assigned to the “Consistently Robust” and the “Improving Frailty” trajectories had severe cognitive impairment at admission, but this prevalence was much higher in those assigned to the other three trajectories. Other characteristics that showed notable differences in the prevalence across trajectory groups included admission source and presence of pain. After “hard-assigning” each resident to their highest probability trajectory, we used multinomial logistic regression models to select the demographic and clinical characteristics to include in the final GBTM. As detailed in the Method Supplement, we found that the magnitude and the direction of association between Alzheimer’s disease or non-Alzheimer’s/other dementia and the assigned physical frailty trajectory groups changed substantially after adjusting for cognitive impairment at admission. This may indicate that cognitive impairment at admission acted as a mediator between Alzheimer’s disease or non-Alzheimer’s/other dementia and physical frailty trajectories. As such, the estimated association between Alzheimer’s disease or non-Alzheimer’s/other dementia and trajectories of physical frailty could be biased when cognitive impairment at admission was included in the same model. Therefore, in the Step 3 GBTM model to assess the association between resident characteristics at admission and the identified physical frailty trajectories over the first six months, the final covariate set included cognitive impairment level at admission, demographic characteristics, and all clinical characteristics except Alzheimer’s disease or non-Alzheimer’s/other dementia. The “Consistently Robust” trajectory was chosen as the reference to highlight the characteristics associated with higher risks to be in the “Improving Frailty”, “Worsening Frailty”, “Consistently Pre-frail”, or “Consistently Frail” trajectories, despite it being the less frequent experience in older nursing home residents.

As shown in Supplement Table S.4a, older residents with worse cognitive impairment at admission had increased odds to follow any of the four trajectories. Notably, those with severe cognitive impairment were 37% more likely to follow the “Improving Frailty” trajectory (aOR: 1.37, 95% CI: 1.27–1.48), twice as likely to follow the “Worsening Frailty” trajectory (aOR: 2.06, 95% CI: 1.93–2.20) and “Consistently Pre-frail” trajectory (aOR: 1.96, 95% CI: 1.85–2.07), and four times as likely to follow the “Consistently Frail” trajectory (aOR: 4.02, 95% CI: 3.81–4.25).

Advancing age and female sex were associated with greater odds of belonging to any of the four trajectories, while those in rural nursing homes had lower odds. Racial/ethnic minority residents had similar odds as non-Hispanic White counterparts to follow the “Improving Frailty” trajectory, and higher odds for the other three trajectories. Compared to those admitted from the community, older adults admitted from acute hospitals had increased odds of belonging to the “Consistently Pre-frail” trajectory (aOR: 2.63, 95% CI: 2.49–2.79), the “Improving Frailty” trajectory (aOR: 4.24, 95% CI: 3.94–4.55) and the “Consistently Frail” trajectory (aOR: 5.48, 95% CI: 5.18–5.80).

Most diagnoses were consistently associated with higher odds of the four trajectories relative to the “Consistently Robust” trajectory. Notably, the aORs for the “Consistently Frail” trajectory were higher than the other three trajectories for cerebrovascular accident/TIA/stroke (aOR: 2.89, 95% CI: 2.67–3.13), multiple sclerosis (aOR: 20.26, 95% CI: 11.39–36.04), Parkinson’s Disease (aOR: 4.91, 95% CI: 4.38–5.50), and hip fracture (aOR: 6.09, 95% CI: 4.36–8.51). Older residents with presence of pain had greater odds of being in the “Consistently Frail” (aOR: 1.80, 95% CI: 1.72–1.89), “Improving Frailty” (aOR: 1.65, 95% CI: 1.55–1.76) and “Consistently Pre-frail” (aOR:1.43, 95% CI: 1.36–1.50) trajectories. Older residents who received antipsychotics or hypnotics in the seven days were less likely to follow the “Improving Frailty”, “Consistently Pre-frail” or “Consistently Frail” trajectories, while those who received antidepressants were more likely to do so.

### Cognitive impairment trajectories in the first six months of nursing home residence and associated characteristics

**Fig. 2:**
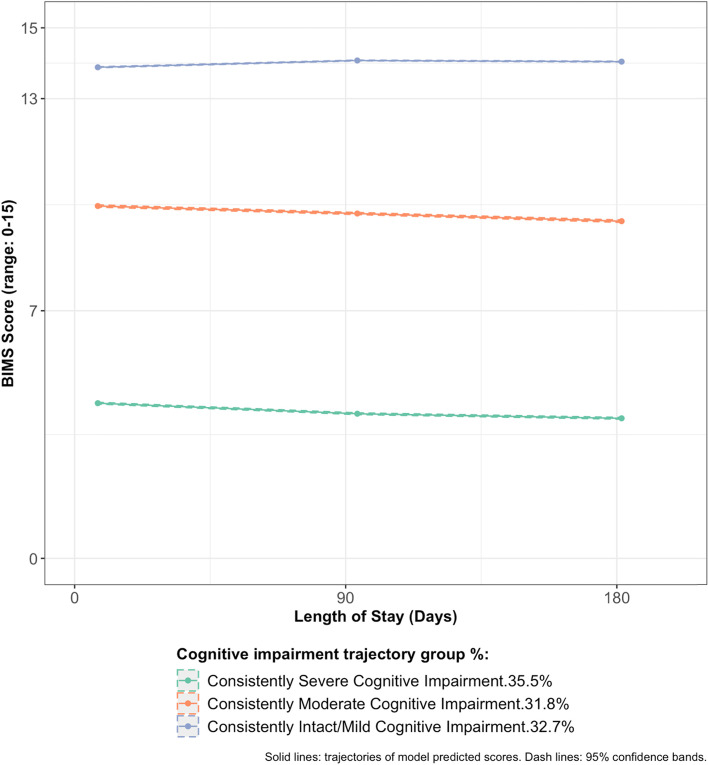
Cognitive impairment trajectories in the first six months of nursing home stay in older adults newly admitted to nursing homes as non-skilled nursing facility residents in 2014–2016

Following a similar model building and selection process (Supplement Table S.2b; Supplement Figure S.2b), we identified the GBTM with three groups as the optimal model for cognitive impairment trajectories. As shown in Fig. 2, for the first six months of post-admission, older residents appeared to follow three distinctive but very consistent trajectories: “Consistently Severe Cognitive Impairment” (35.5%), “Consistently Moderate Cognitive Impairment” (31.8%), and “Consistently Intact/Mild Cognitive Impairment” (32.7%).

Older residents were then assigned to the cognitive impairment trajectory group to which they had the highest posterior probability of belonging (Step 2). At admission, about 57.8% of the older residents assigned to the “Consistently Severe Cognitive Impairment Trajectory” were frail, which was 8% higher than those assigned to the “Consistently Intact/Mild Cognitive Impairment Trajectory”. The distribution of demographic and clinical characteristics are summarized in Supplement Table S.3b. All demographic and clinical characteristics were selected (Method Supplement) for the Step 3 GBTM to examine how at-admission physical frailty, demographic and clinical characteristics were associated with the identified cognitive impairment trajectories over the six months after admission. We used “Consistently Intact/Mild Cognitive Impairment” as the reference trajectory.

As shown in Supplement Table S.4b, older adults who were pre-frail or frail at admission had greater odds of following the “Consistently Moderate Cognitive Impairment” trajectory (pre-frail—aOR: 1.18, 95% CI: 1.14–1.22; frail—aOR: 1.68, 95% CI: 1.62–1.74) and the “Consistently Severe Cognitive Impairment” trajectory (pre-frail—aOR: 1.43, 95% CI: 1.37–1.48; frail—aOR: 2.69, 95% CI: 2.59–2.80).

Older adults with advancing age, from racial/ethnic minority groups, or who were residing in rural nursing homes had greater odds of being in the “Consistently Moderate Cognitive Impairment” or “Consistently Severe Cognitive Impairment” trajectories, while those admitted from acute hospitals or other non-community sources were less likely to do so. Female residents were less likely than males to belong to the “Consistently Moderate Cognitive Impairment” trajectory. There were no sex differences for the “Consistently Severe Cognitive Impairment” trajectory.

Alzheimer’s disease, cerebrovascular accident/TIA/stroke, non-Alzheimer’s/other dementia, seizure disorder/epilepsy or hip fracture were associated with greater odds for the “Consistently Moderate Cognitive Impairment” or “Consistently Severe Cognitive Impairment” trajectories. Conversely, cancer, heart failure, hypertension, diabetes mellitus, multiple sclerosis, Parkinson’s disease, arthritis, osteoporosis, asthma/COPD/chronic lung disease, anxiety disorder, depression or presence of pain were consistently associated with lower odds. Older residents who received antipsychotics or antidepressants were more likely to follow the “Consistently Moderate Cognitive Impairment” or “Consistently Severe Cognitive Impairment” trajectories.

### Dual trajectory of physical frailty and cognitive impairment

**Fig. 3:**
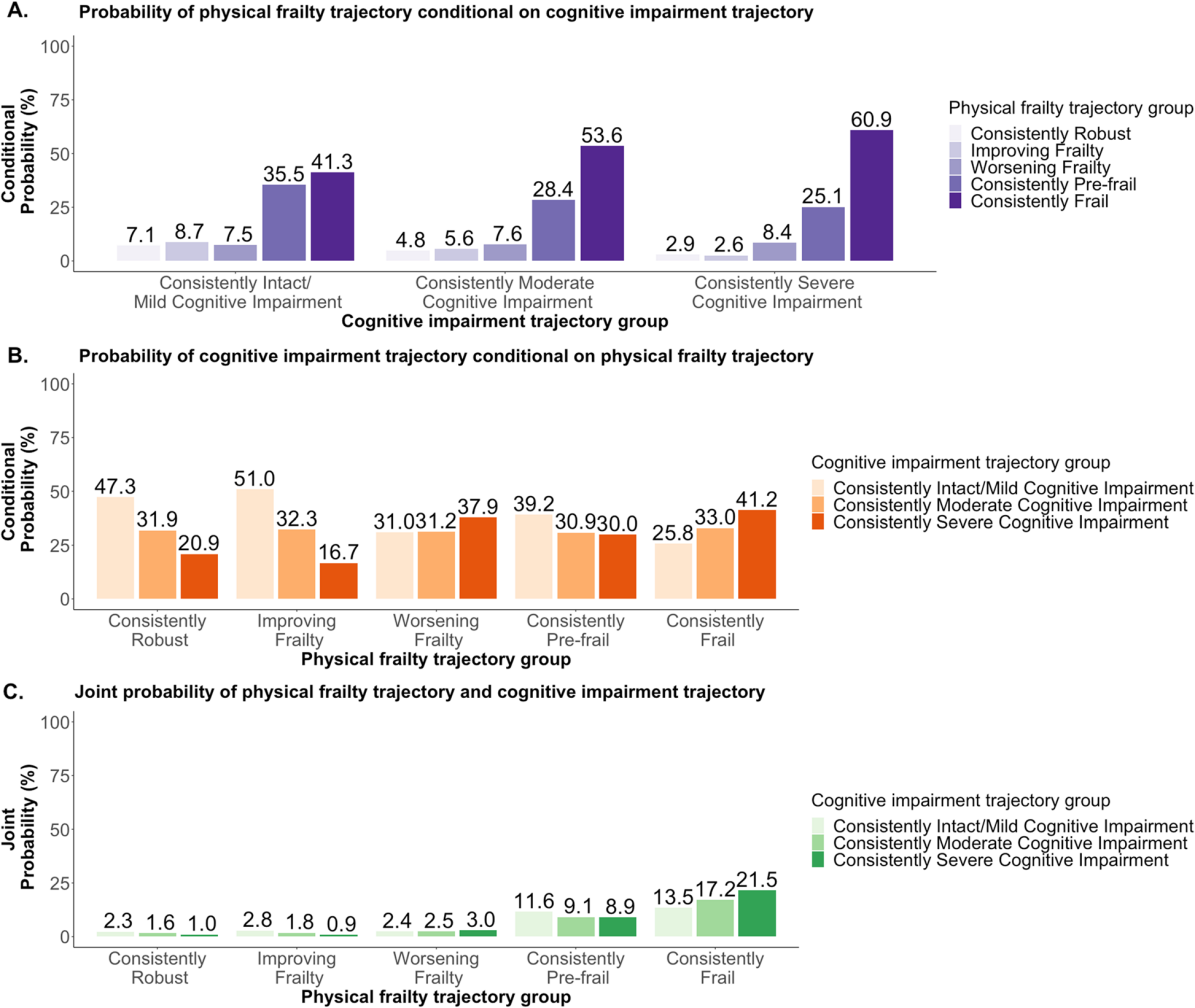
Unadjusted dual trajectory model of physical frailty and cognitive impairment in the first six months of nursing home stay in older adults newly admitted to nursing homes as non-skilled nursing facility residents in 2014–2016

The conditional probabilities linking trajectories of physical frailty and trajectories of cognitive impairment are shown in Fig. 3.

In older residents following a “Consistently Intact/Mild Cognitive Impairment” trajectory, over a third followed the “Consistently Pre-frail” trajectory. The probability of following a “Consistently Frail” trajectory was highest (60.9%) in those with a “Consistently Severe Cognitive Impairment” trajectory.

In residents who followed a “Consistently Robust” or “Improving Frailty” physical frailty trajectory, around half followed a “Consistently Intact/Mild Cognitive Impairment” trajectory. Over 40% of those in the “Consistently Frail” trajectory followed a “Consistently Severe Cognitive Impairment” trajectory.

In their six months after nursing home admission, 21.5% of older residents followed “Consistently Frail” and “Consistently Severely Cognitive Impairment” trajectories.

## Discussion

To the best of our knowledge, this is the first study to quantify the trajectories of physical frailty and cognitive impairment in U.S. nursing home residents on the national level. The use of GBTM with continuous measures of physical frailty and cognitive impairment enabled us to describe the “natural history” of these two prominent conditions, identify the demographic and clinical characteristics associated with the respective trajectories, and examine the relationship between the trajectories of physical frailty and trajectories of cognitive impairment in older adults’ first six months of nursing home residence.

Consistent with prior studies that found heterogeneous progression trajectories in community-dwelling older adults [12], five distinct physical frailty trajectories were identified in older nursing home residents. While most older adults’ experience of physical frailty did not appear to change, 1 in 18 residents showed improvement and 1 in 13 showed worsening over time. With the growing body of studies that have effectively improved older adults’ physical frailty through physical and nutritional interventions [22–25, 27], clinical management individualized to older residents’ respective trajectories may be helpful to maintain their physical robustness, help them improve more steadily, reduce the rate of worsening, or address the factors that contributed to their consistently pre-frail or frail status in the nursing home setting. The demographic and clinical characteristics that were found to be associated with the physical frailty trajectories can be helpful in identifying and triaging distinct subgroups of older residents to proper care.

At-admission physical frailty level appeared to be a strong determinant of older residents’ physical frailty trajectory in the next six months. An exception to this observation was those following the “Improving Frailty” trajectory, who had a baseline predicted FRAIL-NH score comparable to the “Consistently Pre-frail” trajectory. Several characteristics may help distinguish between the two: seizure disorder/epilepsy, other fracture, and presence of pain were associated with higher odds to be in the “Improving Frailty” trajectory than the “Consistently Pre-frail” trajectory. But perhaps the strongest factors to differentiate these two trajectories was severe cognitive impairment, admission from acute hospitals, and age ≥ 85 years.

The experience of older residents while living in the nursing home may influence the trajectory of physical frailty. In addition to nursing home admission [41, 42], social isolation and loneliness are notable risk factors for physical frailty [43, 44] in older adults. The improving trajectory may reflect some older adults’ experience of adjusting to communal living, becoming more engaged in social activities, and having the support from other residents and staff. Our data did not allow us to evaluate the extent to which this possible explanation could be empirically supported. Alternatively, given the medical supervision provided in the nursing home setting, some older residents may experience fewer issues related to access and/or adherence to medications and other non-pharmacological therapies to manage their chronic conditions, which may in turn be reflected in the improving trajectory. Future research is needed to evaluate how such post-admission factors can be associated with physical frailty trajectories to optimize care management for potential improvements in physical frailty in this setting.

The “Intact/Mild Cognitive Impairment” trajectory was consistent with prior research that mostly identified a subgroup of older adults who started with a high cognitive function and remained relatively stable over time [11]. However, unlike previous studies that commonly found trajectories of cognitive decline to various degrees [11, 45], older nursing home residents with moderate and severe cognitive impairment at nursing home admission showed minimal changes during the six months post admission. Aside from the differences in population and setting, this could be attributed to the fact that change in cognitive function is a slow process [11], and therefore six months may not be adequate to capture substantial changes on the trajectories. Additionally, our eligibility criteria excluded those who were unable to participate in the BIMS at all three times. As a result, older residents who may have their cognitive impairment measured by the staff through the Cognitive Performance Scale (CPS) [46] due to extensive cognitive decline were not included in our study. Although the Cognitive Function Scale [47] combines both scales, the combined categories may not capture the continuous changes over time as adequately as a continuous score from one scale. Potential floor effects from the assessment tools in terms of not being able to detect cognitive changes below a certain threshold could also have played a role [11], but there is limited research to thoroughly examine if BIMS suffered from such a disadvantage in the nursing home population.

Comorbid conditions were associated with reduced odds to follow the “Consistently Moderate Cognitive Impairment” or “Consistently Severe Cognitive Impairment” trajectories. However, this finding does not suggest that older adults with greater comorbidities were less likely to follow the more adverse cognitive impairment trajectories. Instead, residents with comorbid conditions likely entered nursing homes for care needs related to dependencies related to activities of daily living and other physical functions and physical illnesses [48, 49], rather than impairment in cognitive function. Older residents who received antipsychotics or antidepressants were more likely to belong to the “Consistently Moderate Cognitive Impairment” or “Consistently Severe Cognitive Impairment” trajectories. Both medications are frequently prescribed to older residents with Alzheimer’s disease or other related dementias to address the behavioral and psychologic symptoms [50, 51]. Exposure to these medications could accelerate cognitive decline [52]; the trajectories identified in our study did not show substantial changes. Because we examined the receipt of psychotropic medications at nursing home admission, future research should consider medication use before and after nursing home admission to evaluate how psychotropic medications could impact cognitive impairment trajectories.

Regarding the relationship between the two sets of trajectories, one previous study in community-dwelling older adults identified four trajectory groups of physical frailty and cognitive impairment, including one marked by the simultaneous accelerated worsening of both conditions which was named “cognitive frailty” [53]. Another study found that in older adults with mild cognitive impairment and mild-moderate Alzheimer’s disease, the progression of physical frailty may be independent of cognitive impairment at its earlier stages and only became positively associated at its later stages [54]. Our study treated physical frailty and cognitive impairment as two separate constructs. This allowed us to focus on the progression trajectories of each individual condition and then describe the correlation between them in nursing home residents. Our study provides longitudinal evidence that the two sets of trajectories were highly correlated. In older nursing home residents who were consistently severely cognitive impaired, three in five were consistently physically frail. This was almost 1.5 times that of those who were consistently cognitive intact or mildly cognitive impaired. For those who were “Consistently Robust”, “Improving Frailty”, and “Consistently Pre-frail”, the majority followed the “Intact/Mild Cognitive Impairment” trajectory. For residents who experienced “Worsening Frailty” or were “Consistently Frail”, the majority followed the “Consistently Severe Cognitive Impairment” trajectory. These findings were similar to a study in older Mexican American community-dwelling adults [55]. However, due to modeling convergence constraints, we were not able to identify relevant resident characteristics associated with both sets of trajectories. Future studies should attempt to incorporate resident characteristics into dual trajectory models, as such analyses would provide further insight on how the progression of physical frailty and cognitive impairment are interrelated over time. As more than 1 in 5 residents remain consistently frail and severely cognitively impaired during the first six months after nursing home admission, more research is needed to examine the risk profiles of these older residents to develop tailored care to address both conditions.

We note a few limitations. We focused on the first six months post admission to nursing home admission. This early period is a critical window when functional impairment could impact older adults’ adjustments to changes in care setting and living environment, as well as long-term health outcomes [28, 56]. We included older adults who resided in nursing homes for 6 months or longer so that we would have sufficient follow-up time. We acknowledge that selection bias may have been introduced. We examined sample characteristics at admission, but some of these characteristics may change over time. Additional research is needed to examine how such changes during older adults’ nursing home residence could alter the identified physical frailty trajectories and cognitive impairment trajectories. We used a validated scale for physical frailty. Nevertheless, the FRAIL-NH is a relatively new scale. The BIMS may not be informative for executive functioning. Items for FRAIL-NH and BIMS were readily available in MDS 3.0, which enabled this work to examine physical frailty at the national level. However, to further our understanding of the trajectories of these two conditions in older adults in nursing homes, additional instruments that could provide a more granular, domain-specific measurement of both conditions in the nursing home setting. Studies using different scales to measure cognitive impairment and physical frailty are warranted.

In conclusion, in older adults’ first six months in a U.S. nursing home, five physical frailty trajectories were identified. While half of older residents were consistently frail, some showed improvement or decline in their physical frailty. Three cognitive impairment trajectories were identified with minimal changes over time. Trajectories of physical frailty were strongly associated with trajectories of cognitive impairment. More than 1 in 5 residents followed the trajectories of being consistently frail and severely cognitive impaired. Older residents may benefit from comprehensive care management approaches tailored to the distinctive trajectories that they were following. The identified sociodemographic and clinical characteristics that were associated with the identified physical frailty and cognitive impairment trajectories can inform our efforts to triage older residents upon admission for proper care.

## Supplementary Information

**Additional file 1: Supplement Figure S.1**. Sample flowchart. **Supplement Figure S.2a.** Graphic depictions of group-based trajectory model with two to six groups for physical frailty. **Supplement Figure S.2b.** Graphic depictions of group-based trajectory model with two to six groups for cognitive impairment. **Supplement Table S.1.** The FRAIL-NH scale. **Supplement Table S.2a.** Fit statistics for trajectory models for physical frailty over the first six months of nursing home stay. **Supplement Table S.2b.** Fit statistics for trajectory models for cognitive impairment over the first six months of nursing home stay. **Supplement Table S.3a.** At-admission cognitive impairment, demographic and clinical characteristics by assigned physical frailty trajectories. **Supplement Table S.3b.** At-admission physical frailty, demographic and clinical characteristics by assigned cognitive impairment trajectories. **Supplement Table S.4a.** Association between demographic and clinical characteristics at admission and physical frailty trajectory groups. **Supplement Table S.4b.** Association between demographic and clinical characteristics at admission and cognitive impairment trajectory groups. **Method Supplement:** Model Building Step 2.

## Data Availability

Restrictions apply to the availability of the data (Minimum Data Set 3.0) under a data use agreement for this study. Minimum Data Set 3.0 is available from www.resdac.org with the permission of the Centers for Medicare and Medicaid Services.
