# Supplementary material for: Trajectories of physical frailty and cognitive impairment in older adults in United States nursing homes
Source: BMC Geriatr. 2022 Apr 19;22:339. doi: 10.1186/s12877-022-03012-8 (PMC9017032; doi:10.1186/s12877-022-03012-8)
Supplement: Supplementary file 1 — Additional file 1: Supplement Figure S.1. Sample flowchart. Supplement Figure S.2a. Graphic depictions of group-based trajectory model with two to six groups for physical frailty. Supplement Figure S.2b. Graphic depictions of group-based trajectory model with two to six groups for cognitive impairment. Supplement Table S.1. The FRAIL-NH scale. Supplement Table S.2a. Fit statistics for trajectory models for physical frailty over the first six months of nursing home stay. Supplement Table S.2b. Fit statistics for trajectory models for cognitive impairment over the first six months of nursing home stay. Supplement Table S.3a. At-admission cognitive impairment, demographic and clinical characteristics by assigned physical frailty trajectories. Supplement Table S.3b. At-admission physical frailty, demographic and clinical characteristics by assigned cognitive impairment trajectories. Supplement Table S.4a. Association between demographic and clinical characteristics at admission and physical frailty trajectory groups. Supplement Table S.4b. Association between demographic and clinical characteristics at admission and cognitive impairment trajectory groups. Method Supplement: Model Building Step 2. [file 12877_2022_3012_MOESM1_ESM.docx]

**Supplement File**

*Yuan Y, et al. Trajectories of physical frailty and cognitive impairment in older adults in United States nursing homes*

| **Titles** | Page |
| --- | --- |
| **Supplement Figure S.1. Sample flowchart** | S-2 |
| **Supplement Figure S.2a. Graphic depictions of group-based trajectory model with two to six groups for physical frailty** | S-3 |
| **Supplement Figure S.2b. Graphic depictions of group-based trajectory model with two to six groups for cognitive impairment** | S-4 |
| **Supplement Table S.1. The FRAIL-NH scale** | S-5 |
| **Supplement Table S.2a. Fit statistics for trajectory models for physical frailty over the first six months of nursing home stay** | S-6 |
| **Supplement Table S.2b. Fit statistics for trajectory models for cognitive impairment over the first six months of nursing home stay** | S-7 |
| **Supplement Table S.3a. At-admission cognitive impairment, demographic and clinical characteristics by assigned physical frailty trajectories** | S-8 |
| **Supplement Table S.3b. At-admission physical frailty, demographic and clinical characteristics by assigned cognitive impairment trajectories** | S-9 |
| **Supplement Table S.4a. Association between demographic and clinical characteristics at admission and physical frailty trajectory groups** | S-10 |
| **Supplement Table S.4b. Association between demographic and clinical characteristics at admission and cognitive impairment trajectory groups** | S-11 |
| **Method Supplement: Model Building Step 2** | S-12 |


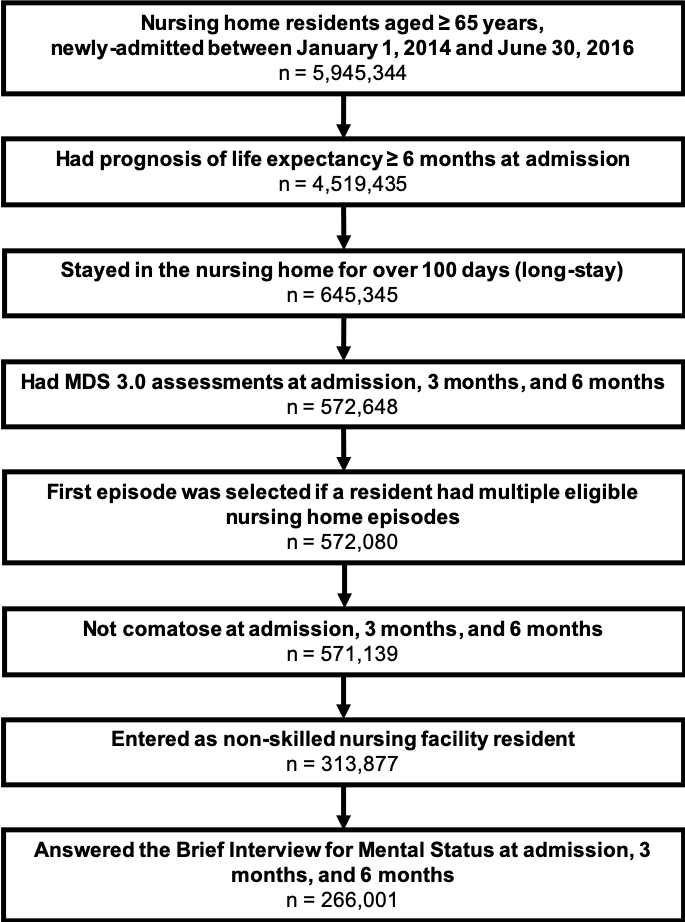


**Supplement Figure S.1. Sample flowchart**

**Supplement Figure S.2a. Graphic depictions of group-based trajectory model with two to six groups for physical frailty**

**
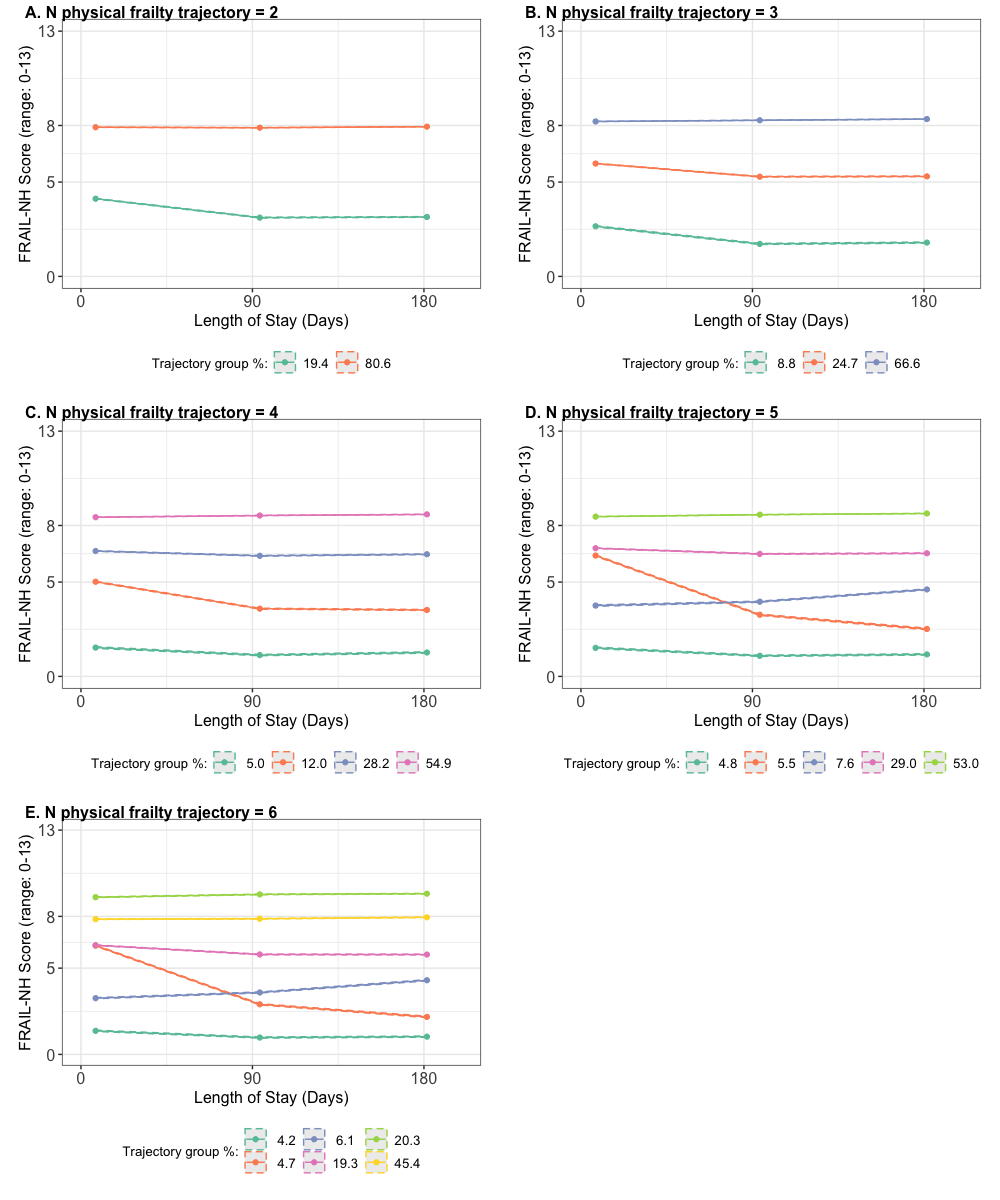
**

**Supplement Figure S.2b. Graphic depictions of group-based trajectory model with two to six groups for cognitive impairment**

**
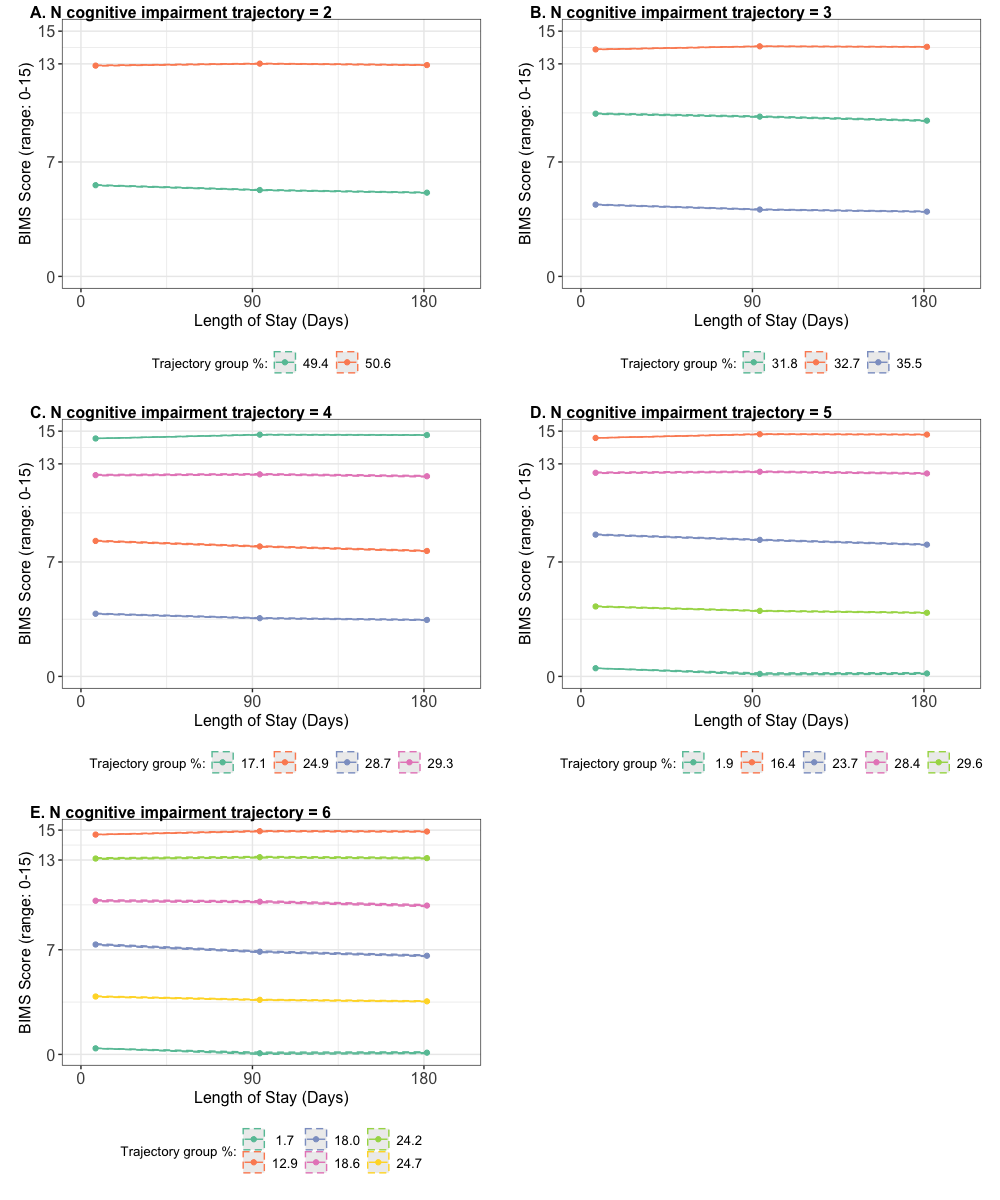
**

**Supplement Table S.1. Items in the FRAIL-NH scale ^1^**

| **Items** | **Item Score** | | | **Items in MDS 3.0** |
| --- | --- | --- | --- | --- |
|  | **0** | **1** | **2** |  |
| **F**atigue ^2^ | No (never or 1 day) | Yes (several days or everyday) | PHQ-9 >= 10 | Section D Mood:  D0300/D0600; D0200-D/D0500-D |
| **R**esistance ^3^ | Independent | With set-up only | Need physical assistance | Section G Functional Status:  G0110B2 |
| **A**mbulation ^4^ | Independent | With assistive device (walker/cane) | Cannot walk | Section G Functional Status:  G0110E1; G0110F1; G0600A; G0600B; G0600C |
| **I**ncontinence | None | Urinary incontinence only | Bowel incontinence | Section H Bladder and Bowel:  H0300; H0400 |
| **L**oss of weight | None | >= 5% in the past 3 months or >= 10% in the past 6 months | n/a ^5^ | Section K Swallowing/Nutritional Status:  K0300 |
| **N**utritional approach | Regular diet | Mechanically altered diet | Require feeding tube | Section K Swallowing/Nutritional Status:  K0500 |
| **H**elp with dressing | Independent | Need help with set up only | Need physical help | Section G Functional Status:  G0110G2 |

Note:

^1^ Kaehr EW, Pape LC, Malmstrom TK, Morley JE. FRAIL-NH predicts outcomes in long term care. J Nutr Health Aging. 2016;20(2):192-198. doi:10.1007/s12603-016-0682-5

^2^ Based on residents’ response to the Patient Health Questionnaire (PHQ-9; MDS 3.0 Section D: Mood).

^3^ Measures if the resident needs assistance to be transferred from one location to another.

^4^ Measures if the resident can walk in a room.

^5^ This item will only receive a score of 0 or 1. A score of 2 is not applicable.

**Supplement Table S.2a. Fit statistics for trajectory models for physical frailty over the first six months of nursing home stay**

| **# Trajectory groups** | **Trajectory shape parameter ^1^** | **BIC** | **Group membership** | **Group AvePP ^2^** | **OCC ^3^** |
| --- | --- | --- | --- | --- | --- |
| 2 | (2 2) | -1525679 | 0.194 (a) | 0.942 (b) | 67.07 |
|  |  |  | 0.806 | 0.981 | 12.55 |
| 3 | (2 2 2) | -1472788 | 0.088 | 0.958 | 235.53 |
|  |  |  | 0.247 | 0.882 | 22.59 |
|  |  |  | 0.666 | 0.951 | 9.71 |
| 4 | (2 2 2 2) | -1453213 | 0.050 | 0.936 | 275.87 |
|  |  |  | 0.120 | 0.888 | 60.44 |
|  |  |  | 0.282 | 0.832 | 12.60 |
|  |  |  | 0.549 | 0.902 | 7.52 |
| **5** | **(2 2 2 2 2)** | **-1441341** | **0.048** | **0.943** | **334.50** |
|  |  |  | **0.055** | **0.838** | **87.48** |
|  |  |  | **0.076** | **0.818** | **54.65** |
|  |  |  | **0.290** | **0.835** | **12.52** |
|  |  |  | **0.530** | **0.908** | **8.61** |
| 6 | (2 2 2 2 2 2) | -1431466 | 0.042 | 0.931 | 307.66 |
|  |  |  | 0.047 | 0.868 | 134.09 |
|  |  |  | 0.061 | 0.838 | 79.70 |
|  |  |  | 0.193 | 0.837 | 21.65 |
|  |  |  | 0.454 | 0.803 | 4.88 |
|  |  |  | 0.203 | 0.780 | 13.96 |

Note: BIC = Bayesian information criterion; AvePP = Average posterior probability; OCC = Odds of correct classification.

^1^ Defines the shape parameters of the trajectory groups: 0 = intercept only, 1 = linear, 2 = quadratic, 3 = cubic.

^2^ Group AvePP of assignment: Based on the maximum probability assignment rule, each individual will be assigned to a group according to the largest posterior probability. For all the individuals assigned to a certain group, an AvePP will be calculated. For each trajectory group, an AvePP of assignment > 0.7 is indicative of good certainty of group assignments.

^3^ OCC: The numerator is the odds of a correct classification into a certain group based on the model, and the denominator is the correct classification into that group based on random assignment, essentially, OCC = [b/(1-b)]/[a/(1-a)]. For each trajectory group, OCC >= 5 suggests high assignment accuracy.

**Supplement Table S.2b. Fit statistics for trajectory models for cognitive impairment over the first six months of nursing home stay**

| **# Trajectory groups** | **Trajectory shape parameter ^1^** | **BIC** | **Group membership** | **Group AvePP** | **OCC** |
| --- | --- | --- | --- | --- | --- |
| 2 | (2 2) | -1836008 | 0.494 (a) | 0.965 (b) | 27.99 |
|  |  |  | 0.506 | 0.949 | 18.14 |
| **3** | **(2 2 2)** | **-1774126** | **0.355** | **0.920** | **20.76** |
|  |  |  | **0.318** | **0.881** | **15.85** |
|  |  |  | **0.327** | **0.945** | **35.10** |
| 4 | (2 2 2 2) | -1751091 | 0.287 | 0.934 | 35.06 |
|  |  |  | 0.249 | 0.858 | 18.10 |
|  |  |  | 0.293 | 0.838 | 12.49 |
|  |  |  | 0.171 | 0.905 | 46.04 |
| 5 | (2 2 2 2 2) | -1739468 | 0.019 | 0.872 | 358.15 |
|  |  |  | 0.296 | 0.885 | 18.25 |
|  |  |  | 0.237 | 0.851 | 18.48 |
|  |  |  | 0.284 | 0.889 | 19.77 |
|  |  |  | 0.164 | 0.897 | 47.01 |
| 6 | (2 2 2 2 2 2) | -1734898 | 0.017 | 0.867 | 389.20 |
|  |  |  | 0.247 | 0.835 | 15.42 |
|  |  |  | 0.180 | 0.780 | 16.16 |
|  |  |  | 0.186 | 0.780 | 15.57 |
|  |  |  | 0.242 | 0.859 | 18.19 |
|  |  |  | 0.129 | 0.852 | 42.03 |

Note: BIC = Bayesian information criterion; AvePP = Average posterior probability; OCC = Odds of correct classification.

^1^ Defines the shape parameters of the trajectory groups: 0 = intercept only, 1 = linear, 2 = quadratic, 3 = cubic.

^2^ Group AvePP of assignment: Based on the maximum probability assignment rule, each resident will be assigned to a group according to the largest posterior probability. For all the individuals assigned to a certain group, an AvePP will be calculated. For each trajectory group, an AvePP of assignment > 0.7 is indicative of good certainty of group assignments.

^3^ OCC: The numerator is the odds of a correct classification into a certain group based on the model, and the denominator is the correct classification into that group based on random assignment, essentially, OCC = [b/(1-b)]/[a/(1-a)]. For each trajectory group, OCC >= 5 suggests high assignment accuracy.

**Supplement Table S.3a. At-admission cognitive impairment, demographic and clinical characteristics by assigned physical frailty trajectories**

|  | | ***Assigned to ^1^…*** | | | | |
| --- | --- | --- | --- | --- | --- | --- |
|  | | **Consistently Robust Trajectory** | **Improving Frailty Trajectory** | **Worsening Frailty Trajectory** | **Consistently Pre-frail Trajectory** | **Consistently Frail Trajectory** |
| *Characteristics at admission* | | (%) | (%) | (%) | (%) | (%) |
| **Cognitive impairment ^2^** | |  |  |  |  |  |
|  | Intact/Mild impairment | 44.7 | 44.4 | 35.0 | 37.3 | 28.6 |
|  | Moderate impairment | 30.9 | 31.0 | 30.0 | 29.6 | 30.4 |
|  | Severe impairment | 24.5 | 24.6 | 34.9 | 33.1 | 41.0 |
| **Age (years)** | |  |  |  |  |  |
|  | 65-<75 | 28.8 | 24.0 | 24.1 | 19.8 | 19.1 |
|  | 75 - <85 | 35.6 | 35.0 | 35.3 | 33.2 | 33.8 |
|  | ≥ 85 | 35.6 | 41.0 | 40.6 | 47.0 | 47.1 |
| **Female** | | 61.1 | 66.4 | 63.8 | 68.1 | 68.0 |
| **Racial/ethnic minority** | | 12.7 | 13.3 | 15.7 | 15.6 | 19.6 |
| **Rural nursing home** | | 44.0 | 34.7 | 34.8 | 27.2 | 21.1 |
| **Admission source** | |  |  |  |  |  |
|  | Community | 54.0 | 38.2 | 53.5 | 40.8 | 28.8 |
|  | Acute hospital | 18.6 | 42.8 | 19.7 | 36.0 | 44.1 |
|  | Other ^3^ | 27.4 | 19.0 | 26.8 | 23.2 | 27.1 |
| **Diagnosis** | |  |  |  |  |  |
|  | Cancer | 5.1 | 6.1 | 5.5 | 5.7 | 5.9 |
|  | Heart failure | 11.9 | 16.0 | 13.2 | 16.5 | 17.4 |
|  | Hypertension | 73.3 | 75.4 | 73.6 | 76.4 | 76.2 |
|  | Diabetes Mellitus | 27.0 | 29.1 | 27.6 | 29.4 | 31.3 |
|  | Alzheimer's Disease | 14.1 | 11.1 | 17.8 | 13.7 | 13.5 |
|  | Cerebrovascular Accident/TIA/Stroke | 6.4 | 7.9 | 7.2 | 9.6 | 14.4 |
|  | Non-Alzheimer’s/other dementia | 39.4 | 37.6 | 45.3 | 40.6 | 43.8 |
|  | Multiple Sclerosis | 0.1 | 0.2 | 0.2 | 0.6 | 0.8 |
|  | Parkinson's Disease | 2.9 | 3.9 | 3.2 | 5.4 | 7.7 |
|  | Seizure disorder/Epilepsy | 4.3 | 4.9 | 4.2 | 4.5 | 5.9 |
|  | Arthritis | 26.3 | 29.5 | 27.0 | 30.4 | 29.3 |
|  | Osteoporosis | 11.8 | 13.1 | 12.8 | 14.2 | 13.7 |
|  | Hip fracture | 0.3 | 1.8 | 0.5 | 2.0 | 3.5 |
|  | Other fracture | 1.5 | 6.6 | 1.8 | 5.9 | 6.5 |
|  | Asthma/COPD/Chronic Lung Disease | 19.0 | 21.3 | 18.0 | 18.4 | 17.8 |
|  | Anxiety disorder | 23.4 | 24.1 | 23.8 | 22.9 | 23.4 |
|  | Depression | 35.6 | 35.6 | 38.0 | 38.5 | 41.5 |
| **Any presence of pain** | | 28.7 | 41.0 | 27.2 | 36.3 | 38.2 |
| **Types of psychotropic medications received** | |  |  |  |  |  |
|  | Antipsychotics | 21.8 | 16.4 | 22.3 | 17.7 | 19.3 |
|  | Antianxiety | 18.1 | 18.5 | 18.0 | 17.9 | 18.8 |
|  | Antidepressant | 41.8 | 42.7 | 45.1 | 45.2 | 48.9 |
|  | Hypnotic | 4.9 | 4.5 | 4.3 | 4.1 | 3.8 |

Note: TIA = transient ischemic attack; COPD = chronic obstructive pulmonary disease

^1^ Older nursing home residents were assigned to the physical frailty trajectories they had the highest posterior probability of belonging to.

^2^ Measured by BIMS using previously validated cutoffs: intact/mild impairment (13-15), moderate impairment (8-12), and severe impairment (0-7).

^3^ Included another nursing home/swing bed, psychiatric hospital, inpatient rehabilitation facility, intellectual disabilities and developmental disabilities facility, long-term care hospitals, hospice, and other unspecified admission sources.

**Supplement Table S.3b. At-admission physical frailty, demographic and clinical characteristics by assigned cognitive impairment trajectories**

|  | | ***Assigned to ^1^…*** | | |
| --- | --- | --- | --- | --- |
|  | | **Consistently Intact/Mild Cognitive Impairment Trajectory** | **Consistently Moderate Cognitive Impairment Trajectory** | **Consistently Severe Cognitive Impairment Trajectory** |
| *Characteristics at admission* | | (%) | (%) | (%) |
| **Physical Frailty ^2^** | |  |  |  |
|  | Robust | 19.0 | 16.9 | 15.7 |
|  | Pre-frail | 31.2 | 28.4 | 26.5 |
|  | Frail | 49.8 | 54.7 | 57.8 |
| **Age (years)** | |  |  |  |
|  | 65-<75 | 31.2 | 18.4 | 12.6 |
|  | 75 - <85 | 33.6 | 33.7 | 34.3 |
|  | ≥ 85 | 35.2 | 47.9 | 53.1 |
| **Female** | | 66.0 | 65.3 | 70.1 |
| **Racial/ethnic minority** | | 15.3 | 17.7 | 19.3 |
| **Rural nursing home** | | 25.6 | 25.8 | 25.4 |
| **Admission source** | |  |  |  |
|  | Community | 30.8 | 34.5 | 10.9 |
|  | Acute hospital | 42.6 | 41.1 | 33.4 |
|  | Other ^3^ | 26.6 | 24.4 | 25.7 |
| **Comorbidities** | |  |  |  |
|  | Cancer | 6.7 | 6.0 | 4.8 |
|  | Heart failure | 21.0 | 17.3 | 11.9 |
|  | Hypertension | 78.0 | 77.2 | 72.9 |
|  | Diabetes Mellitus | 36.4 | 30.9 | 24.2 |
|  | Alzheimer's Disease | 3.4 | 10.7 | 25.5 |
|  | Cerebrovascular Accident/TIA/Stroke | 12.4 | 12.9 | 10.3 |
|  | Non-Alzheimer’s/other dementia | 19.3 | 43.7 | 61.5 |
|  | Multiple Sclerosis | 1.1 | 0.5 | 0.2 |
|  | Parkinson's Disease | 7.2 | 7.0 | 4.9 |
|  | Seizure disorder/Epilepsy | 5.6 | 5.6 | 4.8 |
|  | Arthritis | 32.4 | 29.2 | 26.8 |
|  | Osteoporosis | 13.2 | 13.5 | 14.1 |
|  | Hip fracture | 2.4 | 2.8 | 2.6 |
|  | Other fracture | 6.7 | 6.1 | 4.7 |
|  | Asthma/COPD/Chronic Lung Disease | 24.2 | 18.4 | 12.9 |
|  | Anxiety disorder | 23.6 | 21.8 | 24.4 |
|  | Depression | 40.5 | 39.9 | 39.1 |
| **Any presence of pain** | | 52.2 | 36.9 | 21.0 |
| **Types of psychotropic medications received** | |  |  |  |
|  | Antipsychotics | 13.0 | 17.0 | 25.9 |
|  | Antianxiety | 18.5 | 16.6 | 20.0 |
|  | Antidepressant | 46.4 | 46.6 | 47.6 |
|  | Hypnotic | 5.7 | 3.7 | 2.8 |

Note: TIA = transient ischemic attack; COPD = chronic obstructive pulmonary disease

^1^ Older nursing home residents were assigned to the physical frailty trajectories they had the highest posterior probability of belonging to.

^2^ Measured by FRAIL-NH using previously validated cutoffs: robust (0-5), pre-frail (6-7), and frail (≥8).

^3^ Included another nursing home/swing bed, psychiatric hospital, inpatient rehabilitation facility, intellectual disabilities and developmental disabilities facility, long-term care hospitals, hospice, and other unspecified admission sources. ^4^ Included non-Alzheimer’s dementia (e.g., vascular or multi-infarct dementia), mixed dementia; frontotemporal dementia (e.g., Pick’s disease), and dementia related to stroke, Parkinson’s or Creutzfeldt-Jakob diseases.

**Supplement Table S.4a. Association between demographic and clinical characteristics at admission and physical frailty trajectories ^1^**

| ***Characteristics at admission*** | | | **Physical frailty trajectories**  ***(ref: Consistently Robust)*** | | | | | | | |
| --- | --- | --- | --- | --- | --- | --- | --- | --- | --- | --- |
|  |  |  | **Improving Frailty** | | **Worsening Frailty** | | **Consistently Pre-frail** | | **Consistently Frail** | |
|  |  |  | aOR | 95% CI | aOR | 95% CI | aOR | 95% CI | aOR | 95% CI |
| **Cognitive impairment (ref: Intact/Mild impairment) ^2^** | | |  |  |  |  |  |  |  |  |
|  | Moderate impairment | | 1.17 | (1.09-1.25) | 1.29 | (1.21-1.37) | 1.21 | (1.15-1.27) | 1.81 | (1.72-1.90) |
|  | Severe impairment | | 1.37 | (1.27-1.48) | 2.06 | (1.93-2.20) | 1.96 | (1.85-2.07) | 4.02 | (3.81-4.25) |
| **Age (ref: 65-<75 years)** | | |  |  |  |  |  |  |  |  |
|  | 75 - <85 | | 1.28 | (1.19-1.38) | 1.16 | (1.08-1.24) | 1.42 | (1.35-1.50) | 1.62 | (1.53-1.71) |
|  | ≥ 85 | | 1.66 | (1.53-1.79) | 1.34 | (1.25-1.44) | 2.24 | (2.11-2.37) | 2.82 | (2.66-2.98) |
| **Female (vs. Male)** | | | 1.19 | (1.12-1.27) | 1.07 | (1.02-1.13) | 1.25 | (1.20-1.31) | 1.30 | (1.24-1.36) |
| **Racial/ethnic minority (ref: Non-Hispanic White)** | | | 0.95 | (0.87-1.03) | 1.29 | (1.20-1.39) | 1.15 | (1.08-1.23) | 1.46 | (1.37-1.55) |
| **Rural nursing home (ref: Urban nursing home)** | | | 0.74 | (0.70-0.79) | 0.66 | (0.63-0.69) | 0.50 | (0.48-0.52) | 0.36 | (0.34-0.37) |
| **Admission source (ref: Community)** | | |  |  |  |  |  |  |  |  |
|  | Acute hospital | | 4.24 | (3.94-4.55) | 0.97 | (0.90-1.04) | 2.63 | (2.49-2.79) | 5.48 | (5.18-5.80) |
|  | Other ^3^ | | 1.05 | (0.97-1.13) | 1.01 | (0.96-1.08) | 1.13 | (1.08-1.19) | 2.21 | (2.10-2.32) |
| **Diagnosis (ref: without the diagnosis)** | | |  |  |  |  |  |  |  |  |
|  | Cardiovascular/metabolic | |  |  |  |  |  |  |  |  |
|  |  | Heart failure | 1.28 | (1.18-1.39) | 1.15 | (1.07-1.25) | 1.40 | (1.31-1.49) | 1.58 | (1.49-1.69) |
|  |  | Hypertension | 1.02 | (0.95-1.09) | 1.01 | (0.95-1.07) | 1.07 | (1.02-1.12) | 0.99 | (0.95-1.04) |
|  |  | Diabetes Mellitus | 1.15 | (1.08-1.23) | 1.11 | (1.04-1.17) | 1.26 | (1.20-1.32) | 1.45 | (1.38-1.52) |
|  | Neurological | |  |  |  |  |  |  |  |  |
|  |  | Cerebrovascular Accident/TIA/Stroke | 1.32 | (1.18-1.47) | 1.17 | (1.06-1.30) | 1.62 | (1.49-1.76) | 2.89 | (2.67-3.13) |
|  |  | Multiple Sclerosis | 3.20 | (1.58-6.50) | 2.74 | (1.39-5.41) | 9.81 | (5.49-17.53) | 20.26 | (11.39-36.04) |
|  |  | Parkinson’s Disease | 1.87 | (1.61-2.17) | 1.13 | (0.97-1.32) | 2.56 | (2.28-2.87) | 4.91 | (4.38-5.50) |
|  |  | Seizure disorder/Epilepsy | 1.41 | (1.24-1.61) | 0.99 | (0.87-1.12) | 1.19 | (1.07-1.31) | 1.56 | (1.41-1.72) |
|  | Musculoskeletal | |  |  |  |  |  |  |  |  |
|  |  | Arthritis | 1.16 | (1.08-1.23) | 1.02 | (0.96-1.08) | 1.20 | (1.14-1.26) | 1.19 | (1.14-1.25) |
|  |  | Osteoporosis | 1.03 | (0.95-1.13) | 1.04 | (0.96-1.12) | 1.08 | (1.01-1.15) | 1.06 | (0.99-1.13) |
|  |  | Hip fracture | 3.30 | (2.31-4.73) | 0.94 | (0.59-1.52) | 3.61 | (2.58-5.07) | 6.09 | (4.36-8.51) |
|  |  | Other fracture | 3.34 | (2.80-3.97) | 0.87 | (0.69-1.10) | 3.03 | (2.58-3.56) | 3.14 | (2.67-3.68) |
|  | Cancer | | 1.19 | (1.06-1.35) | 1.13 | (1.01-1.27) | 1.14 | (1.04-1.25) | 1.26 | (1.15-1.39) |
|  | Asthma/COPD/Chronic Lung Disease | | 1.12 | (1.05-1.21) | 1.00 | (0.93-1.06) | 0.99 | (0.94-1.04) | 1.00 | (0.95-1.06) |
|  | Mental health | |  |  |  |  |  |  |  |  |
|  |  | Anxiety disorder | 1.08 | (1.00-1.18) | 0.99 | (0.92-1.07) | 0.95 | (0.90-1.01) | 0.90 | (0.85-0.96) |
|  |  | Depression | 1.00 | (0.92-1.08) | 1.07 | (1.00-1.15) | 1.12 | (1.06-1.19) | 1.23 | (1.16-1.30) |
| **Any presence of pain (ref: no presence of pain)** | | | 1.65 | (1.55-1.76) | 1.00 | (0.94-1.06) | 1.43 | (1.36-1.50) | 1.80 | (1.72-1.89) |
| **Types of psychotropic medications received** | | |  |  |  |  |  |  |  |  |
|  | Antipsychotics | | 0.72 | (0.66-0.77) | 0.99 | (0.93-1.05) | 0.78 | (0.74-0.82) | 0.75 | (0.72-0.79) |
|  | Antianxiety | | 1.07 | (0.98-1.17) | 0.98 | (0.91-1.06) | 1.05 | (0.98-1.12) | 1.13 | (1.06-1.21) |
|  | Antidepressant | | 1.17 | (1.08-1.26) | 1.14 | (1.06-1.22) | 1.19 | (1.13-1.27) | 1.40 | (1.32-1.48) |
|  | Hypnotic | | 0.85 | (0.75-0.97) | 0.90 | (0.80-1.01) | 0.85 | (0.77-0.93) | 0.81 | (0.73-0.89) |

Note: TIA = transient ischemic attack; COPD = chronic obstructive pulmonary disease; aOR = adjusted odds ratio; CI = confidence interval.

^1^ Multinomial logistic model with the identified physical frailty trajectories as the dependent variable, cognitive impairment as the main independent variable, adjusting for all other covariates in this table.

^2^ Measured by BIMS using previously validated cutoffs: intact/mild impairment (13-15), moderate impairment (8-12), and severe impairment (0-7).

^3^ Included another nursing home/swing bed, psychiatric hospital, inpatient rehabilitation facility, intellectual disabilities and developmental disabilities facility, long-term care hospitals, hospice, and other unspecified admission sources.

**Supplement Table S.4b. Association between demographic and clinical characteristics at admission and cognitive impairment trajectory groups ^1^**

| ***Characteristics at admission*** | | | **Cognitive impairment trajectories**  ***(ref: Consistently intact/mild cognitive impairment)*** | | | |
| --- | --- | --- | --- | --- | --- | --- |
|  |  |  | **Consistently Moderate Cognitive Impairment** | | **Consistently Severe Cognitive Impairment** | |
|  |  |  | **aOR** | **95% CI** | **aOR** | **95% CI** |
| **Physical frailty (ref: Robust) ^2^** | | |  |  |  |  |
|  | Pre-Frail | | 1.18 | (1.14-1.22) | 1.43 | (1.37-1.48) |
|  | Frail | | 1.68 | (1.62-1.74) | 2.69 | (2.59-2.80) |
| **Age (ref: 65-<75 years)** | | |  |  |  |  |
|  | 75 - <85 | | 1.71 | (1.66-1.77) | 2.37 | (2.28-2.46) |
|  | ≥ 85 | | 2.75 | (2.65-2.84) | 4.42 | (4.25-4.59) |
| **Female (vs. Male)** | | | 0.86 | (0.83-0.88) | 1.03 | (1.00-1.06) |
| **Racial/ethnic minority (ref: Non-Hispanic White)** | | | 1.43 | (1.39-1.48) | 1.80 | (1.74-1.87) |
| **Rural nursing home (ref: Urban nursing home)** | | | 1.14 | (1.11-1.17) | 1.19 | (1.16-1.23) |
| **Admission source (ref: Community)** | | |  |  |  |  |
|  | Acute hospital | | 0.96 | (0.93-0.99) | 0.68 | (0.65-0.70) |
|  | Other ^3^ | | 0.79 | (0.76-0.81) | 0.65 | (0.63-0.67) |
| **Comorbidities (ref: without the comorbid condition)** | | |  |  |  |  |
|  | Cardiovascular/metabolic | |  |  |  |  |
|  |  | Heart failure | 0.85 | (0.83-0.88) | 0.63 | (0.61-0.66) |
|  |  | Hypertension | 0.94 | (0.91-0.97) | 0.79 | (0.77-0.81) |
|  |  | Diabetes Mellitus | 0.90 | (0.88-0.92) | 0.71 | (0.69-0.73) |
|  | Neurological | |  |  |  |  |
|  |  | Alzheimer's Disease | 4.97 | (4.68-5.27) | 17.66 | (16.70-18.67) |
|  |  | Cerebrovascular Accident/TIA/Stroke | 1.21 | (1.17-1.26) | 1.05 | (1.01-1.10) |
|  |  | Non-Alzheimer’s/other dementia ^4^ | 4.00 | (3.88-4.11) | 9.88 | (9.59-10.17) |
|  |  | Multiple Sclerosis | 0.62 | (0.53-0.71) | 0.35 | (0.29-0.43) |
|  |  | Parkinson's Disease | 0.86 | (0.81-0.90) | 0.49 | (0.46-0.52) |
|  |  | Seizure disorder/Epilepsy | 1.19 | (1.13-1.26) | 1.06 | (1.00-1.12) |
|  | Musculoskeletal | |  |  |  |  |
|  |  | Arthritis | 0.82 | (0.79-0.84) | 0.70 | (0.68-0.72) |
|  |  | Osteoporosis | 0.96 | (0.92-0.99) | 0.92 | (0.88-0.95) |
|  |  | Hip fracture | 1.27 | (1.18-1.37) | 1.50 | (1.38-1.63) |
|  |  | Other fracture | 1.04 | (0.99-1.09) | 1.00 | (0.94-1.06) |
|  | Cancer | | 0.92 | (0.88-0.97) | 0.78 | (0.74-0.83) |
|  | Asthma/COPD/Chronic Lung Disease | | 0.82 | (0.80-0.85) | 0.62 | (0.60-0.64) |
|  | Mental health | |  |  |  |  |
|  |  | Anxiety disorder | 0.92 | (0.89-0.95) | 0.92 | (0.89-0.96) |
|  |  | Depression | 0.95 | (0.92-0.98) | 0.78 | (0.75-0.81) |
| **Any presence of pain (ref: no presence of pain)** | | | 0.60 | (0.59-0.62) | 0.32 | (0.31-0.32) |
| **Types of psychotropic medications received** | | |  |  |  |  |
|  | Antipsychotics | | 1.37 | (1.32-1.42) | 1.99 | (1.92-2.07) |
|  | Antianxiety | | 1.01 | (0.97-1.05) | 1.22 | (1.17-1.27) |
|  | Antidepressant | | 1.12 | (1.08-1.16) | 1.21 | (1.17-1.25) |
|  | Hypnotic | | 0.71 | (0.67-0.75) | 0.52 | (0.48-0.56) |

Note: TIA = transient ischemic attack; COPD = chronic obstructive pulmonary disease; aOR = adjusted odds ratio; CI = confidence interval.

^1^ Multinomial logistic model with the identified cognitive impairment trajectories as the dependent variable, cognitive impairment as the main independent variable, adjusting for all other covariates in this table.

^2^ Measured by FRAIL-NH using previously validated cutoffs: robust (0-5), pre-frail (6-7), and frail (≥8).

^3^ Included another nursing home/swing bed, psychiatric hospital, inpatient rehabilitation facility, intellectual disabilities and developmental disabilities facility, long-term care hospitals, hospice, and other unspecified admission sources. ^4^ Included non-Alzheimer’s dementia (e.g., vascular or multi-infarct dementia), mixed dementia; frontotemporal dementia (e.g., Pick’s disease), and dementia related to stroke, Parkinson’s or Creutzfeldt-Jakob diseases.

**Method Supplement: Model Building Step 2**

The goal of this step was to select older nursing home residents’ demographic and clinical characteristics to be included in the final group-based trajectory model to assess their associations with the trajectories of physical frailty or trajectories of cognitive impairment. In the following, we explained this variable selection process for the model on physical frailty trajectory.

First, older nursing home residents were assigned to the physical frailty trajectory group with the highest posterior probability.

For each demographic characteristic, we examined its crude associations with the assigned trajectories, which were all statistically significant; and then the adjusted associations when all demographic characteristics were adjusted for, which showed minimal changes and remained significant. Hence, all demographic characteristics were selected.

For all clinical characteristics, the crude and adjusted odds ratios adjusting for all demographic characteristics were statistically significant, indicating their association with the assigned physical frailty trajectories. However, given the study objective, cognitive impairment at admission would be included in the model. So, we further compared the adjusted associations adjusting for demographic characteristics only and the adjusted associations adjusting for demographic characteristics and cognitive impairment at admission, to explore if and how cognitive impairment would impact the association between each clinical characteristic and the physical frailty trajectories. We used 10% change in the odds ratios and identified three clinical characteristics that showed substantial changes: Alzheimer’s disease, Non-Alzheimer’s/Other dementia, and any presence of pain. Moreover, the direction of the associations between Alzheimer’s disease or Non-Alzheimer’s/Other dementia with assigned consistently pre-frail trajectory and the assigned consistently frailty trajectory flipped. Based on clinical knowledge, for Alzheimer’s disease or Non-Alzheimer’s/Other dementia, it was likely that cognitive impairment acted as a mediator between these two conditions and physical frailty trajectories, so adjusting for it would bias their association. For pain, on the other hand, it would be more likely that the presence of cognitive impairment influenced older residents’ assessment of pain, making cognitive impairment a confounder, and adjusting for it would not bias the association between pain and physical frailty trajectories. Therefore, Alzheimer’s disease and Non-Alzheimer’s/Other dementia were not included in the final model for physical frailty trajectory.

A very similar process was carried out to select the demographic and clinical characteristics for cognitive impairment trajectories, the only difference being that cognitive impairment at admission was replaced by physical frailty at admission. All demographic and clinical characteristics were significantly associated with the assigned cognitive impairment trajectories. When comparing the adjusted associations between each clinical characteristic with the assigned cognitive impairment trajectories adjusting for demographic characteristics only versus adjusting for demographic characteristics and physical frailty at admission, no characteristic showed a >10% change, so all were included in the final model for cognitive impairment trajectory.
